# Advances in pig models of human diseases

**DOI:** 10.1002/ame2.12223

**Published:** 2022-03-27

**Authors:** Naipeng Hou, Xuguang Du, Sen Wu

**Affiliations:** ^1^ College of Animal Science and Technology China Agricultural University Beijing China; ^2^ Sanya Institute of China Agricultural University Sanya China; ^3^ State Key Laboratory of Agrobiotechnology, College of Biological Sciences China Agricultural University Beijing China

**Keywords:** animal model, gene‐editing, human disease, pig

## Abstract

Animal models of human diseases play a critical role in medical research. Pigs are anatomically and physiologically more like humans than are small rodents such as mice, making pigs an attractive option for modeling human diseases. Advances in recent years in genetic engineering have facilitated the rapid rise of pig models for use in studies of human disease. In the present review, we summarize the current status of pig models for human cardiovascular, metabolic, neurodegenerative, and various genetic diseases. We also discuss areas that need to be improved. Animal models of human diseases play a critical role in medical research. Advances in recent years in genetic engineering have facilitated the rapid rise of pig models for use in studies of human disease. In the present review, we summarize the current status of pig models for human cardiovascular, metabolic, neurodegenerative, various genetic diseases and xenotransplantation.

## Graphical abstract

Animal models of human diseases play an important role in the research of medical field. In the current review, we attempt to summarize the advances of the pig models for human cardiovascular diseases, metabolic diseases, neurodegenerative diseases, and some other genetic diseases. We also discuss the areas that need to be improved.

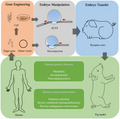

## 1 INTRODUCTION

Research on human disease pathogenesis is critical for progress in therapeutic medicine. Insufficient sample acquisition, environmental conditions, and ethics often impede studies to examine human disease directly, and therefore animal models are crucial for gaining in vivo insight into disease etiology and pathogenesis. Mice and other small rodents have long been important model animals for basic research, and have contributed greatly to our understanding of human disease pathogenesis. However, the limitations of rodent models are many. For example, metabolic rate is influenced by body size, and their small size leads to difficulties in performing surgery and using organs (Table 1). Considerable differences exist between rodents and humans in the regulatory networks controlling the activity of the immune system, metabolic functions, and responses to stress.^1^, ^2^ For example, age‐associated fasting blood glucose exhibits differential trends between mice and monkeys/humans.^3^ Importantly, more than 80% of potential therapeutics fail in human trials despite showing safety and efficacy in mice.^4^

**TABLE 1:** General features of experimental animals^6^, ^7^, ^8^, ^9^, ^10^, ^11^

Species	Average body length (cm)	Average body weight (kg)	Average age (year)	Pregnancy length (day)	Offspring per litter	Heart as % of body weight	Brain as % of body weight
*Homo sapiens*	170	40–100	72	280	1–2	0.5	2.1
*Mus musculus*	8–10	0.03–0.05	1–2	18–21	3–12	0.5	1.42
*Rattus norvegicus*	17	0.2–0.6	1–2	20–23	6–12	0.38	0.29
*Oryctolagus cuniculus*	48	3.5–7.5	5–12	23–34	3–9	0.3	0.4
*Sus scrofa*	125	40–120	20	114	10	0.6	0.5
*Ovis aries*	90	80–100	10–15	142–155	1–2	0.27	0.12
*Bos taurus*	220	500–900	20–23	270	1	0.03	0.08
*Canis familiaris*	75	10–25	12–15	58–67	5–6	0.85	0.59
*Rhesus monkey*	50	8–10	20	150	1–2	0.36	0.9

Pigs are one of the most common domestic animals in the world. Compared to other livestock and primates, pigs have a rapid growth rate, short generation intervals, large litter sizes, and standardized breeding techniques. These advantages, combined with comparable human and pig body sizes, anatomical and physiological characteristics, diets, and genome (Table 1),^5^ have driven a gradual rise in the use of pigs as animal models for human diseases.

Similar body and organ sizes between pigs and humans will likely hasten the translation of pig studies (in comparison to mouse studies) to the clinic. Even before the advent of transgenic and gene‐editing technology, pig models enabled important advances in human heart, bone, metabolism, and even genetic diseases, to name a few. For example, pig models of acute myocardial infarction (MI) were generated by permanently ligating the trunk near one‐third of the apex after the first branch or by inflating an angioplasty balloon in the mid‐left anterior descending artery,^12^, ^13^, ^14^, ^15^, ^16^facilitating testing, and development of MI therapies for use in humans. Similarly, bone and cartilage models have been generated through surgically‐induced lesions in pigs for the development of biomaterials.^17^, ^18^ As both humans and pigs are monogastric omnivores, diet modification has been a fruitful approach for creating pig models of human metabolic disease. A high‐fat diet (HFD) induces obesity and metabolic syndrome and has been used in pigs to research the renal disease and nonalcoholic fatty liver disease.^19^, ^20^ To obtain genetic disease models, ENU chemical mutagenesis has been used to induce a set of point mutations that frequently mimic the subtlety and heterogeneity of human genetic lesions.^21^ For example, microphthalmia‐associated transcription factor (*MITF* ^+/l247s^) mutants mimic Waardenburg’s syndrome type II, dual oxidase 2 (*DUOX 2* ^D409G/D409G^) mutants mimic congenital hypothyroidism, SRY‐box transcription factor 10 (*SOX 10* ^+/R109W^) mutants mimic Mondini dysplasia, and mutants with a 2 bp CC insertion in the melanocortin receptor 1 (*MC1R*) mimic albinism.^22^, ^23^, ^24^, ^25^ These genetic models are heritable and require no special diet or surgical intervention to obtain experimental animals (Table 2).

**TABLE 2:** Pig models by surgery, HFD and ENU mutagenesis

Human disease	Method	Phenotype	References
Myocardial infarction	Permanent ligation of the trunk near one‐third of the apex after the first branch	Mir‐590‐3p suppresses proliferation and migration of cardiac fibroblasts	^13^
Myocardial infarction	Inflated angioplasty balloon in the mid‐left anterior descending artery for 90‐min	Reduction of apoptosis by Cortical bone stem cells	^16^
Myocardial infarction	90‐min occlusion of the left anterior coronary artery	Improvement of cardiomyocyte proliferation by microrna‐199a	^48^
Meniscal lesions	4 mm defect created in the medial meniscus by surgery	Reduced the chondral lesions by tissue‐engineered construct	^17^
Cartilage lesions	6 mm created on the femoral condyles of stifle joints by surgery	Repaired by living hyaline cartilaginous graft	^18^
Renal disease	High‐fat diet	Diabetic changes and glomerulomegaly	^20^
Nonalcoholic fatty liver disease	High‐fat diet	Selenoproteins against damage induced by high‐fat diet	^19^
Waardenburg’s syndrome type II	ENU mutagenesis	Hearing loss, white coat color and *MITF* ^+/L247S^	^22^
Congenital hypothyroidism	ENU mutagenesis	Anemia, immunodeficiency and *DUOX 2* ^D409G/D409G^	^23^
Mondini dysplasia	ENU mutagenesis	Inner ear mondini malformation and *SOX 10* ^+/R109W^	^22^
Albinism	ENU mutagenesis	White coat color and 2 bp CC insertion in the *MC1R*	^25^

With the development of transgenic and gene‐editing technology, genetically engineered pig models are greatly expanding our understanding of human disease pathogenesis while aiding the development of novel treatments. Existing pig models comprise a wide range of human diseases, including cardiovascular diseases, diabetes, neurodegenerative diseases, genetic diseases, and cancer. Our review will focus on important genetically engineered pig models of human diseases in current use, generated using novel approaches, such as the combined technologies of microinjection (MI), somatic cell nuclear transfer (SCNT), and embryo transfer. We include helpful references for the construction of pig models and the research of human diseases.

## 2 CURRENT PIG MODELS OF HUMAN DISEASE

### 2.1 Metabolic diseases

Metabolic diseases are diseases that disrupt the normal metabolic process and are generally affected by both genetics and environments. Common metabolic diseases include obesity, hyperglycemia, hyperlipidemia, hypertension, hyperuricemia, fatty liver, cardiovascular disease, and cerebrovascular disease.

#### 2.1.1 Diabetes

Diabetes mellitus (DM) is a group of metabolic disorders characterized by high blood sugar. Prolonged high blood glucose can damage the kidneys, heart, eyes, and nervous system. The three main classifications of DM are type I, type II, and gestational diabetes, although rarer forms of diabetes caused by mutations in specific genes also occur. Although type I and type II diabetes can appear in individuals without any family history of diabetes, they still show a highly heritable and generally involve insulin (INS) deficiency (type I) or insulin resistance (type II).^26^ As insulin is secreted by the pancreatic islet cells, pigs—with a pancreas similar in size, shape, and blood circulation to the human pancreas—have become an attractive diabetes model. *INS* is believed to play a central role in insulin‐dependent diabetes, permanent neonatal diabetes, type 10 juvenile mature diabetes, and hyperinsulinemia. Mutations^27^ and deletions^28^ of *INS* were achieved in pigs using transgenic and gene editing techniques, providing invaluable models for studying the onset of diabetes and insulin supplement therapy. These pig models are often improved by insulin treatment and can be used for the research of insulin supplementation and islet transplantation. Type II diabetes is mainly caused by insufficient insulin secretion and excessive insulin resistance. In 2010, Renner et al.^29^ generated transgenic pigs expressing a dominant‐negative GIP (glucose‐dependent insulinotropic polypeptide) receptor (GIPR[dn]) in pancreatic islets, demonstrating an essential role of GIP^30^ for insulin secretion, the proliferation of β‐cells, and physiological expansion of β‐cell mass. As patients with type II diabetes show significant insulin resistance to exogenous GIP, these pigs are good models to study the role of GIP in glucose homeostasis and pancreatic development. IAPP can induce oxidative stress and further promote the production of amyloid deposits. Its deposition is considered to be one of the major causes of type II diabetes. Zou et al.^31^ successfully established an *IAPP* gene humanized pig model, which exhibited symptoms of human type II diabetes, such as increased glucose tolerance. These pigs are suitable models for research into islet amyloid deposits in type II diabetes. In addition to the two main types of diabetes, Umeyama et al.^32^ generated cloned pigs with a mutation in human hepatocyte nuclear factor 1α (*HNF‐1α*), which has been reported to cause type III maturity‐onset diabetes of the young (MODY3).^33^ Although the majority of cloned MODY3 pigs died two weeks after birth, the viable pigs, showed high blood glucose levels and proved useful for studying the disease.

Following the development of gene‐editing technology, researchers also pay attention to models with multiple gene modifications. In 2015, Kong et al.^34^ developed knock‐in pigs using the polycistronic system, which contains an expression cassette of 11‐β‐hydroxysteroid dehydrogenase 1 (*11β‐HSD1*) and another expression cassette of human islet amyloid polypeptide (*HIAPP*) and C/EBP homologous protein (*CHOP*). 11β‐HSD1 is important in insulin resistance when hIAPP and CHOP can induce β cell apoptosis in the pancreas. These pigs showed diabetic phenotypes such as hepatic insulin resistance and pancreatic cell apoptosis, which modeled type II diabetes better than some pigs with single‐gene modifications. Similarly, Zhang et al.^35^ engineered pigs to carry three knock‐in risk genes, glucose‐dependent insulinotropic polypeptide receptor (*GIPR* ^*dn*^), human islet amyloid polypeptide (*hIAPP*), and Patatin‐like phospholipase domain‐containing three variant rs738409 C>G p.I148M (*PNPLA3* ^*I148M*^), resulting in glucose and lipid metabolism disorders, abnormal fat development and liver necrosis, ideal for research on non‐alcoholic fatty liver disease (NAFLD) and type II diabetes.

#### 2.1.2 Atherosclerosis

Atherosclerosis promotes cardiovascular disease, and lipid metabolism disorder is the pathological basis of atherosclerosis. Therefore, understanding abnormal lipid metabolism, such as high blood lipid, high cholesterol, and obesity, is vital.^36^, ^37^ Atherosclerosis is usually characterized by the deposition of lipids, cholesterol, and sugar complexes beginning from the intima and histiocytosis, leading to calcification.^38^ Low‐density lipoprotein and apolipoprotein are closely related to blood lipid levels and have therefore been a focus of atherosclerosis research. In 2013, al‐Mashhadi et al.^39^ generated proprotein convertase subtilisin/kexin type 9 (*PCSK* 9) mutation pigs, which exhibited reduced low‐density lipoprotein receptor (LDLR) levels and developed severe hypercholesterolemia and spontaneous atherosclerosis. Similarly, in 2014, Davis et al.^40^ inserted a neomycin‐resistance cassette (NeoR) into the pig *LDLR* gene, disrupting its normal expression. In addition to spontaneous development of certain features of human atherosclerosis, atherosclerosis in LDLR mutant pig models could be accelerated by placing pigs on high‐fat and high‐cholesterol diets. The *PCSK 9* transgenic pigs and the *LDLR* knockout pigs both focus on the regulation of low‐density lipoprotein to model human hypercholesterolemia. However, there is currently no evidence to prove that PCSK 9(D374Y) is functionally important in pigs. Compared to the *PCSK 9* transgenic pigs, the LDLR^−/−^ pigs have a shortened time to development of atherosclerosis. Focusing on apolipoprotein, a pig model of hypertriglyceridemia was developed in 2012 by Wei et al.,^41^ who targeted apolipoprotein (Apo) CIII, a key apolipoprotein in triglyceride metabolism. The pigs expressed human *ApoCIII* in the liver and intestinal tract. However, human *ApoC* *III* transgenic pigs are still the preferred tools for studying the mechanisms of hypertriglyceridemia‐associated diseases and for potential drug development, and it was unclear whether these pigs developed atherosclerosis. In 2018, Fang et al.^42^ generated apolipoprotein E (*ApoE*) knockout pigs in which severe hypercholesterolemia and human‐like atherosclerotic lesions could be induced by a high‐fat, high‐cholesterol diet. The rate of cholesterol elevation under a high‐fat diet in *ApoE* ^−/−^ pigs is higher than in the *PCSK9* transgenic pigs and the *LDLR* ^−/−^ pigs, and the hypertriglyceridemia phenotype was found in *ApoE* ^−/−^ pigs but not the *PCSK 9* transgenic pigs or the *LDLR* ^−/−^ pigs, suggesting that *ApoE* ^−/−^ pigs may be a better model to simulate human atherosclerosis. Advances in gene‐editing technology led Huang et al.^43^ to create *ApoE* and *LDLR* double gene knockout pigs in 2017. These pigs had significantly increased serum levels of low‐density lipoprotein cholesterol (LDL‐C) and total cholesterol (TC) and enriched the available models. Besides LDL, some cholesterol absorption relevant genes also influence the development of atherosclerosis. In 2015, Wang et al.^44^ generated a pig model with InDels of *NPC1L1*, an important gene in cholesterol absorption.

In addition to abnormal lipid metabolism, atherosclerosis can be caused by abnormal glucose metabolism.^45^ In 2017, Yang et al.^46^ used zinc finger nuclease technology to create *PPARγ* mono‐allelic knockout pigs, which proved to be a good model for both atherosclerosis and type 2 diabetes. These pig models provide new research opportunities for early asymptomatic human atherosclerosis and other cardiovascular diseases that are difficult to study and treat.

#### 2.1.3 Myocardial infarction

Myocardial infarction (MI) is a major cause of morbidity and mortality worldwide. Atherosclerosis is a risk factor for MI, as the rupture of atherosclerotic plaques leads to thrombus and sudden obstruction of the coronary artery, further resulting in myocardial ischemic necrosis. Various pig models of cardiovascular disease have been widely used in the development of treatments. In 2019, Hobby et al.^16^ guided an angioplasty balloon through the femoral artery to the mid‐LAD past the first diagonal branch. The MI model generated by inflation of the balloon led to the discovery that cortical bone stem cells (CBSCs) influence cardiomyocyte and noncardiomyocyte cell death and immune cell recruitment in the heart following MI.^47^ MicroRNAs have proven to be another rewarding avenue for MI research. MiR‐590‐3p was shown to suppress proliferation, migration, and differentiation of cardiac fibroblasts, whereas^13^ MiR‐144‐3p and microRNA‐199a appear to induce these cardiac fibroblast programs.^12^, ^48^ At present, most research models of myocardial infarction are disposable models prepared by surgery, which have limitations for long‐term use. If a stable genetic model can be developed in the future, the research on myocardial infarction will be greatly accelerated (Table 3).

**TABLE 3:** Pig models of metabolic diseases

Human disease	Gene	Modification	References
Mody3	*HGF*	Mutation	^32^
Type 2 diabetes	*GIPR*	Mutation	^29^
Diabetes, coronary heart disease	*PPARγ*	Knockout	^46^
Permanent neonatal diabetes mellitus	*INS*	Mutation	^27^
Type 2 diabetes	*11β‐HSD 1, HIAPP, CHOP*	Knock‐in	^34^
Diabetes	*INS*	Knock‐in	^28^
Type 2 diabetes	*hIAPP*	Knockout	^31^
NAFLD	*GIPR* ^*dn*^ *, hIAPP, PNPLA3* ^*I148M*^	Knock‐in	^35^
Hypertriglyceridemia	*ApoC*III	Knock‐in	^41^
Hypercholesterolemia, atherosclerosis	*PCSK9*	Mutation	^39^
Hypercholesterolemia, atherosclerosis	*LDLR*	Knock‐in	^40^
Disorder of cholesterol absorption	*NPC1L1*	Knockout	^44^
Serum LDL‐C and TC levels increase	*ApoE and LDLR*	Knockout	^43^
Hypercholesterolemia, atherosclerosis	*ApoE*	Knockout	^42^

### 2.2 Neurodegenerative diseases

Neurodegenerative diseases are functional disorders caused by the loss of neurons and/or their myelin sheaths in the brain and spinal cord. The most common diseases include Alzheimer’s disease (AD), Parkinson’s disease (PD), Huntington’s disease (HD), and amyotrophic lateral sclerosis (ALS).

#### 2.2.1 Alzheimer’s disease

Alzheimer’s disease, accounting for approximately 50%~80% of human dementia cases,^49^ is a neurodegenerative disease with hidden onset, characterized by general dementia such as memory impairment, aphasia, executive dysfunction, and personality behavior changes. Patients usually exhibit accumulation of extracellular amyloid‐beta (Aβ) to form senile plaques and intracellular neurofibrillary tangles of microtubule‐binding protein Tau in the gray matter of the brain. At present, amyloid precursor protein (*APP*), presenilin 1 (*PSEN1*), and presenilin 2 (*PSEN2*) are considered to be pathogenic genes of familial AD. In 2009, Kragh et al.^50^ generated an AD pig model of transgenic human *APP695sw*. Although high expression of the transgene was detected in different brain regions of this pig model, there was no elevated Aβ level in tissues or memory impairment in 1‐year‐old pigs.^51^ In 2013, Jakobsen et al.^52^ used recombinase‐mediated cassette exchange (RMCE) technology to generate a *PSEN1* ^*M146I*^ mutant pig model. AD pigs carrying both *APP* ^*695sw*^ and *PSEN 1* ^*M146I*^ mutations were subsequently generated in 2016. These pigs were found to accumulate Aβ‐42 in their brains^53^ at around 10–18 months. Several known pathogenic genes of familial AD have been modified in pig models. Also, AD pigs carrying triple mutations of *hAPP (K670N/M671L, I716V, and V717I)*, *hTau (P301L)*, and *hPS1 (M146V and L286P)* were generated using the polycistronic vector system. These pigs were similarly found to accumulate Aβ‐40 and Aβ‐42 in their brain,^54^ a significant phenotype of AD patients.

#### 2.2.2 Parkinson’s disease

Parkinson’s disease, also known as paralysis tremors, is a neurodegenerative disease caused by the degeneration of dopamine neurons in the substantia nigra and the presence of Lewy bodies in the neurons.^55^, ^56^ In 2014, Yao et al.^57^ generated *DJ‐1* gene knockout pigs using TALEN. Although the expression of DJ‐1 was inhibited at the protein level, defective cloning led to the early death of these animals. In 2014, Zhou et al.^58^ generated a *PARK2* and *PINK1* double knockout pig with deficient protein levels of both gene products, and in 2016, Wang et al.^59^ generated pigs with triple gene knockouts of *DJ‐1*, *Parkin*, and *PINK1* using CRISPR/Cas9. In 2018, Zhu et al.^60^ developed *SCNA* knock‐in pigs carrying three missense mutations (E46K, H50Q, and G51D) known to cause Parkinson’s disease. No typical symptoms of PD have been observed in any of these pig models, possibly because PD is a progressive disease that occurs mostly in the elderly.

#### 2.2.3 Huntington’s disease

Huntington’s disease is a rare autosomal dominant genetic disorder. Due to variations in Huntington protein (HTT), patients typically develop motor symptoms, cognitive dysfunction, and mental disorders. In 2010, Yang et al.^61^ generated HD pigs with *HTT* mutations that suffered significant involuntary movements. In 2018, Yan et al.^62^ found that endogenous expression of full‐length HTT mutants in pigs elicited significant neuronal degeneration, which effectively mimics human Huntington’s disease. This single gene mutation has resulted in the current pig models that simulate Huntington’s disease well. Future use of these models to search for effective treatments will be an important application of these pig models (Table 4).

**TABLE 4:** Pig models of neurodegenerative diseases

Human disease	Gene	Modification	References
Alzheimer	*APP695sw*	Knock‐in	^50^
Alzheimer	*PSEN1* ^*MI46I*^	Mutation	^52^
Alzheimer	*APP* ^*SW*^ *, PSEN1* ^*MI46I*^	Mutation	^53^
Alzheimer	*hAPP, hTau, hPS1*	Mutation	^54^
Huntington	*HTT*	Mutation	^61^
Huntington	*HTT*	Knock‐in	^62^
Parkinson	*Parkin, DJ‐1*	Knockout	^57^
Parkinson	*PARK2, DJ‐1, PINK1*	Knockout	^59^
Parkinson	*SNCA*	Knock‐in	^60^
Parkinson	*PARK2, PINK1*	Knockout	^58^

### 2.3 Genetic diseases

Genetic diseases generally refer to diseases caused by changes in genetic material or disease genes. In addition to the metabolic diseases and neurodegenerative diseases discussed above, pig models of cystic fibrosis, Duchenne muscular dystrophy, hemophilia, and various cancers have also been developed for medical research.

#### 2.3.1 Cystic fibrosis

Cystic fibrosis (CF), a recessive genetic disease with a single gene mutation, is caused by dysfunction of the CF transmembrane conductance regulator (*CFTR*). The disease starts in early childhood and affects many tissues and organs, including the respiratory tract, lungs, gastrointestinal tract, pancreas, liver, reproductive tract, and sweat glands. Due to defective chloride ion channels in CF patients, respiratory mucus gland secretions become dehydrated and viscous, resulting in respiratory tract infection, airway obstruction, and meconium obstruction. Viscous secretions can additionally block the reproductive system, leading to male infertility.^63^, ^64^ The pig model of cystic fibrosis is an outstanding example of a genetically engineered pig as a model of human disease. In 2008, a pig model with the *CFTR* allele deletion and another with the most common mutation (ΔF508) were generated by Rogers et al., using a recombinant adeno‐associated virus (RAAV) delivery system.^65^ While approximately 15% of CF patients are born with meconium blocking, meconium blocking rates were 100% in *CFTR* ^−/−^ pigs, and a little bit less in *CFTR* ^+/ΔF508^ pigs. Subsequent studies have shown that *CFTR* ^ΔF508/ΔF508^ pigs develop meconium blocking, abnormal pancreatic and bile secretion,^66^ and lung diseases similar to those of CF patients, which develop spontaneously within a few weeks of birth.^67^ Based on studies of the *CFTR* ^−/−^ pigs, Stoltz et al.^68^ established a corrected model for intestinal expression in 2013, which successfully alleviated meconium obstruction. Thus, the *CFTR* ^−/−^ pig models replicate most of the features of human CF and have shown tremendous promise for translational therapies.^69^

#### 2.3.2 Duchenne muscular dystrophy

Muscular dystrophy is a genetic disorder characterized by progressive muscle weakness, wasting, and muscle degeneration. These diseases mainly include Duchenne muscular dystrophy (DMD), Becker muscular dystrophy (BMD), limb‐girdle muscular dystrophy (LGMD), congenital muscular dystrophy (CMD), and Emery‐Dreifuss Muscular dystrophy (EDMD).^70^, ^71^ DMD is an incurable X‐linked genetic disease caused by deletion, point mutation, or duplication of the *DMD* gene.^72^ Patients tend to die in their 20s or 30s due to weaknesses in the muscles of the heart and lungs. In 2013, Klymiuk et al.^73^ used gene targeting and SCNT to generate a pig model with a deletion of Exon 52 of *DMD*. This pig model developed symptoms similar to human DMD patients, for instance, elevated serum creatine kinase activity, myofibrosis, and loss of myotrophin. However, use of the DMD pig model has been greatly restricted by the considerable rates of pig neonatal death. Yu et al.^74^ used CRISPR/Cas9 gene‐editing technology to accurately edit exon 27 of *DMD*, generating another DMD pig model in 2016. This model also displayed a phenotype similar to human DMD with loss of myotrophic protein and myocardial damage. However, similar to the previous model, these pigs are prone to premature death. Moretti et al.^75^ found that a truncated *DMD* ^Δ51–52^ pig model improved skeletal muscle function and heart rhythm as well as reducing neonatal death, and recent studies by Chiappalupi et al.^76^ found that injection of porcine Sertoli cells can eliminate the inflammatory response and the expression of dystrophin. Overall, DMD is a disease with single gene mutations. Pig DMD models hold great promise in the development of drugs and treatments for DMD.

#### 2.3.3 Cancer

Carcinoma is the most common type of malignant tumor originating from epithelial tissue. In 2010, Luo et al.^77^ reported a pig model with a knockout of the breast cancer‐associated gene (*BRCA1*) mediated by adenovirus. Although the *BRCA1* ^+/∆11^ pigs were able to develop to term, they had high perinatal mortality. No one pig survived more than 18 days, leading the model to be adjusted further. In 2012, Flisikowska et al.^78^ produced abnormal lesions and adenomas in large intestines of pigs by mutating *adenomatous polyposis coli* (*APC*) at sites 1311 and 1016. In these pigs, a single allele mutation of *APC* was sufficient to initiate the well‐characterized precancer sequence leading to growths similar to those in patients with familial adenomatous polyposis in human colorectal lesions, which has not been possible in the mouse models. *RUNX 3* is considered to be a tumor suppressor gene associated with gastric adenocarcinoma. In 2016, Kang et al.^79^ established a pig model with a *RUNX 3* knockout, providing opportunities for gastric cancer research. In 2016, Saalfrank et al.^80^ generated a targeted *TP53* knockout pig, which developed osteosarcoma in the long bone, skull, and mandible. Some genes tend to cause more than one type of cancer. In 2014, Sieren et al.^81^ generated pigs with a mutant *TP53* gene that developed multiple tissue lesions such as lymphoma, Wilm’s neuroblastoma, and bone‐derived tumor. In 2015, Schook et al.^82^ constructed a pig model that could be conditionally induced to express various tumor types via mutation of *KRAS* ^*G12D*^ and *TP53* ^*R167H*^ via Cre recombinase expression. In 2017, Wang et al.^83^ used TALEN and SCNT techniques to produce pigs simulating human non‐small cell lung cancer (NSCLC). These pigs achieved time–space and site‐specific expression of the mutant proteins by Cre induction of rearrangement of echinoderm microtubule‐associated protein 4 (EML4) and anaplastic lymphoma kinase (ALK) genes. This inducible system may be used to study many other cancers.

#### 2.3.4 Other genetic diseases

Additional pig models have been developed to recapitulate various other genetic diseases over the years. Von Willebrand disease is an inherited hemorrhagic disorder generally caused by an autosomal dominant plasma *vWF* deficiency. In 2014, Hai et al.^84^ generated a *vWF* knockout pig model of von Willebrand disease, which showed significant prolonged bleeding and defective coagulation.

Hemophilia comprises a group of recessive X‐linked inherited clotting disorders in patients lacking various clotting factors. Hemophilia B is caused by lack of factor IX (*F9*) gene. In 2020, Chen et al.^85^ reported that targeted pig knockouts lacking a functional *F9* gene showed obvious symptoms of hemophilia B, such as cruor disorder, synovitis, and cartilage destruction. Moreover, the symptoms were significantly rescued by knocking the human *F9* gene into the knockout pigs. This research suggests new ways to correct hemophilia B in the future by genome editing.

Hutchinson‐Gilford progeria syndrome (HGPS) is a rare genetic disorder that often causes premature aging and cardiovascular complications. Introducing heterozygous mutations of the *LMNA* gene into pigs induced growth retardation, lipodystrophy, skin and bone changes, cardiovascular disease, and death in adolescence.^86^ The mean lifespan of these pigs is just about 6 months, making them good models for longevity studies in clinics.

Loss‐of‐function mutations in the *COL2A1* gene are the etiology of type II collagenopathy. *COL2A1* mutant pigs exhibit bone dysplasia and tracheal collapse, modeling aspects of human spondyloepiphyseal dysplasia and stickler syndrome type I.^87^

Waardenburg’s disease is a syndrome of deafness, white hair, and eye disease. Wang et al.^88^ generated *MITF* mutant pigs using CRISPR/Cas9, which also developed white fur and hearing impairments. Then in 2021, Yao et al.^89^ successfully rescued anophthalmia and hearing loss in the cloned pigs using single‐stranded oligodeoxynucleotide (ssODN) and long donor plasmid DNA as the repair template.

Another epidermal disorder, oculocutaneous albinism type I was modeled in pigs by either *TYR* gene fragment knockout or point mutation.^58^, ^90^ The pigs completely lost dark pigment in skin, hair, and eyes, showing visible signs of the disease, but this model is still worth further analysis.

Unlike mice, pigs have a high cone density and dense photoreceptor retinal area, similar to humans. Cloned pigs with a rhodopsin (*Rho*) mutation showed reduced light sensitivity, similar to patients with inherited retinal degeneration^91^, ^92^, ^93^; *ELOVL4* mutant pigs, which simulate Stargardt disease type 3, showed photoreceptor loss and reduced retinal response.^94^

Hereditary tyrosinemia type I (HT1) is caused by a deficiency of fumaryl acetoacetic acid hydrolase (FAH), which leads to liver failure. Hickey et al.^95^ generated *FAH* ^+/−^ cloned pigs with an adeno‐associated virus‐mediated gene targeting strategy. The *FAH* ^−/−^ offspring showed severe liver damage, but unlike humans, FAH‐deficiency in pigs causes a lethal defect in utero, and interestingly the defect of FAH could be cured by 2‐(2‐nitro‐4‐trifluoromethylbenzoyl)‐1,3 cyclohexanedione (NTBC).

Phenylketonuria, caused by a deficiency of phenylalanine hydroxylase (PAH), can lead to neurocognitive impairment, behavioral problems, eczema, and hypopigmentation. Koppes et al.^96^ generated a pig model of phenylketonuria with symptoms including hyperphenylalaninemia, growth retardation, hypoplasia, ventricular dilation, and decreased gray matter volume. But they did not show devastating neurocognitive and neurological clinical characteristics.

In summary, pig models have been widely used to simulate human diseases, and most genetic diseases can be studied by preparing pig models. Especially when the causal gene in humans is known (Table 5).

**TABLE 5:** Pig models of genetic diseases

Human disease	Gene	Modification	References
Cystic fibrosis	*CFTR*	Knockout, mutation	^64^
Cystic fibrosis	*CFTR*	Knockout	^67^
Cystic fibrosis	*CFTR*	Knockout	^68^
Duchenne muscular dystrophy	*DMD*	Knockout	^73^
Duchenne muscular dystrophy	*DMD*	Knockout	^74^
Breast cancer	*BRCA1*	Knockout	^77^
Colorectal cancer	*APC* ^*1311*^ *, APC* ^*1016*^	Mutation	^78^
Lymphoma, wilm‐blastoma, and bone tumors	*TP53* ^*R167H*^	Mutation	^81^
Cancer	*KRAS* ^*G12D*^ *, TP53* ^*R167H*^	Mutation	^82^
Gastric cancer	*RUNX3*	Knockout	^79^
Osteosarcoma	*TP53*	Knockout	^80^
Lung cancer	*EML4, ALK*	Knock‐in	^83^
Von Willebrand disease	*vWF*	Knockout	^84^
Hemophilia B	*hF9*	Knock‐in	^85^
Hutchinson‐Gilford progeria syndrome	*LMNA*	Mutation	^86^
Waardenburg’s	*MITF*	Knockout	^88^
Ocular skin albinism type 1	*TYR*	Knockout	^58^
Retinitis pigmentosa	*Rho*	Mutation	^91^
Retinitis pigmentosa	*Rho*	Mutation	^92^
Retinitis pigmentosa	*Rho*	Mutation	^93^
Stargardt disease type 3 (STGD 3)	*ELOVL4*	Knockout, mutation	^94^
Tyrosinemia type I	*FAH*	Knockout	^95^
Phenylketonuria	*PAH*	Knockout	^96^

### 2.4 Xenotransplantation

One of the most important roles of pigs in the biomedical field is as tissue and organ donors. There is currently a serious shortage of life‐saving tissues and organs for human clinical transplantation. The structure and function of organs are similar between pigs and humans. Because of this, pigs have attracted great interest in the field of xenotransplantation. Corneas, hearts, kidneys, livers, lungs, nerve cells, and islets of pigs have been studied as candidates for xenotransplantation.

One of the key problems in xenotransplantation is immune rejection. The presence of α‐1,3‐galactose (α‐Gal) epitopes on pig cells is a major obstacle to successful xenotransplantation. α‐galactosyl transferase 1 (*GGTA 1*) is an important gene involved in the biosynthesis of α‐1,3‐galactose. Researchers have established *GGTA 1* knockout or mutant pig models.^97^, ^98^, ^99^, ^100^, ^101^ Similarly, N‐glycolylneuraminic acid (NeuGc) is a non‐Gal xenoantigen in pigs which can compromise successful transplantation to human hosts. This challenge was met by the establishment of a CMP‐Neu5Ac hydroxylase (*CMAH*) knockout pig model.^102^, ^103^ Since immune rejection is often not controlled by a single gene, researchers have also generated a combined knockout of GGTA 1 and CMAH, as well as some other xenoantigen genes such as iGb3S and β4 GalNT2.^104^, ^105^, ^106^, ^107^ In addition to xenoantigens, major histocompatibility complex class I (MHC I)^108^, ^109^, ^110^ and NK cells^111^ are important factors in host immunity, for which pig models have been established to address potential problems. Furthermore, the establishment of several pig models with severe combined immunodeficiency and inactivation of porcine endogenous retroviruses has reduced concerns about the spread of zoonotic diseases and has provided important materials for the advancement of xenotransplantation.^112^, ^113^, ^114^, ^115^, ^116^, ^117^

Solid organ xenotransplantation between pig and non‐human primates is also a key research priority before human clinical trials. In recent years, with the development of xenotransplantation, several types of solid organ xenotransplantation have been tested in non‐human primates with some success, including heart,^118^, ^119^ kidney,^120^ lung,^121^ and liver.^122^ Even more exciting, the world’s first gene‐edited pig heart transplant into a human was carried out in January 2022. Although the patient died unfortunately after two months, this is still a milestone in the search for a solution to the shortage of human organs. Almost at the same time, the world’s first pig kidney transplant into a human was reported.^123^ We expect that in the future, gene‐modified pigs will certainly provide new opportunities for the shortage of human organs (Table 6).

**TABLE 6:** Pig models of xenotransplantation

Human disease	Gene	Modification	References
Immunological rejection (αGal)	*GGTA1*	Knockout, mutation	97, 98, 99, 100, 101
Immunological rejection (non‐Gal)	*CMAH*	Knockout	102, 103
Immunological rejection	*GGTA1, CMAH*	Knockout	105, 106
Immunological rejection	*GGTA1, CMAH, iGb3S*	Knockout	^104^
Immunological rejection	*GGTA1, β4GalNT22, CMAH*	Knockout	^107^
Immunological rejection (MHC I)	*SLA*	Knockout	^110^
Immunological rejection (MHC I)	*B2M*	Knockout	108, 109
Immunological rejection (NK cell)	*ULBP1*	Knockout	^111^
Severe combined immunodeficiency	RAG2	Knockout	^117^
Severe combined immunodeficiency	RAG1/2	Knockout	^116^
Severe combined immunodeficiency	*RAG2, IL2RG*	Knockout	^113^
Severe combined immunodeficiency	*IL2RG*	Knockout	112, 114
Inactivation of porcine endogenous retroviruses	*PERV*	Knockout	^115^

## 3 CONCLUSION

Currently, there are pig models for a variety of human diseases including cardiovascular, metabolic, neurodegenerative, and other genetic diseases, which have provided considerable support for the analysis and treatment of human diseases. Recently, the COVID‐19 pandemic caused by severe acute respiratory syndrome coronavirus 2 (SARS‐CoV‐2) has led to a serious global public health crisis. The analysis of the pathogenesis of infection, the development of diagnostic and therapeutic methods, and the validation of vaccine and drug products all require large animal models similar to human clinical pathogenesis. Du et al.^124^ replaced pig angiotensin‐converting enzyme 2 (*ACE2*) by site‐specific knock‐in of human hACE2 and found that primary epithelial cells isolated from the lungs and kidneys of this humanized pig model were highly sensitive to SARS‐CoV‐2 infection. In conclusion, pig models have great potential to advance the study of human diseases, from the study of pathogenesis to the development and utilization of drugs, and even as tissue and organ donors.

In addition, there is much that needs improving in pig gene editing, in vitro embryo culture, and assisted reproduction. In recent years, research on pig pluripotent stem cells has also provided new opportunities for the production of cloned pigs. Although the emergence of gene‐editing technology has greatly accelerated progress in pig models for studying genetic background and for testing drugs, therapeutics, and methods of delivery, safety, and ethical issues cannot be ignored. On the one hand, humans and pigs are different in many ways, and drugs and treatments developed in pig models must be determined to be safe before clinical tests. On the other hand, because of the existence of zoonosis, care must be taken at every stage of the experiment to avoid cross‐contamination and the spread of disease. Apart from safety and ethical issues, animal welfare also affects society’s willingness to condone animal research. The health of the animal used as a model is not only critical to obtaining reliable results but is also a responsibility for every researcher. Improving the nutrition, physical environment, health, behavioral interactions, and mental state of pigs will promote the development and social acceptance of pig models.^125^ By addressing the importance of these issues, pig models will continue to be an important source of support for the advancement of human medicine in the future.

## CONFLICT OF INTEREST

The authors declared no conflicts of interest.

## AUTHOR CONTRIBUTIONS

Naipeng Hou conceived and wrote the original draft of the manuscript. Xuguang Du and Sen Wu revised the manuscript. All authors critically read and contributed to the manuscript, and approved its final version.
